# Network signatures link hepatic effects of anti-diabetic interventions with systemic disease parameters

**DOI:** 10.1186/s12918-014-0108-0

**Published:** 2014-09-11

**Authors:** Thomas Kelder, Lars Verschuren, Ben van Ommen, Alain J van Gool, Marijana Radonjic

**Affiliations:** 1 TNO, Research Group Microbiology & Systems Biology, Zeist, The Netherlands; 2 Department of Laboratory Medicine, Radboud University Nijmegen Medical Center, Nijmegen, The Netherlands; 3 Faculty of Physics, Mathematics and Informatics, Radboud University Nijmegen, Nijmegen, The Netherlands; 4 Current address: EdgeLeap B.V, Utrecht, The Netherlands

**Keywords:** Network biology, Type II diabetes mellitus, Metabolic health, Transcriptomics, Systems biology

## Abstract

**Background:**

Multifactorial diseases such as type 2 diabetes mellitus (T2DM), are driven by a complex network of interconnected mechanisms that translate to a diverse range of complications at the physiological level. To optimally treat T2DM, pharmacological interventions should, ideally, target key nodes in this network that act as determinants of disease progression.

**Results:**

We set out to discover key nodes in molecular networks based on the hepatic transcriptome dataset from a preclinical study in obese LDLR-/- mice recently published by Radonjic et al. Here, we focus on comparing efficacy of anti-diabetic dietary (DLI) and two drug treatments, namely PPARA agonist fenofibrate and LXR agonist T0901317. By combining knowledge-based and data-driven networks with a random walks based algorithm, we extracted network signatures that link the DLI and two drug interventions to dyslipidemia-related disease parameters.

**Conclusions:**

This study identified specific and prioritized sets of key nodes in hepatic molecular networks underlying T2DM, uncovering pathways that are to be modulated by targeted T2DM drug interventions in order to modulate the complex disease phenotype.

## 1 Background

To improve our understanding and ability to intervene with complex multifactorial diseases such as type 2 diabetes mellitus (T2DM) it is important to investigate the molecular networks underlying the biological system and elucidate which and how interactions within this system contribute to pathology [1]. This will enable discovery of novel therapeutic pathways that trigger a specific cascade of processes underlying pathology development and subsequently optimally target a wide range of disease parameters. This is a challenging task, since disease networks are large and complex, involving many disease-driving processes which are in turn composed of tens of thousands interconnected components (e.g. genes, proteins, metabolites) and hundreds of thousands possible functional interactions [2].

Large-scale experiments and curation efforts provide a large map of possible molecular pathways, protein interactions and transcription factor targets [3]-[7]. Such networks have been shown to provide mechanistic insights and identify key regulators in various disease settings. For example, integration of different experimental data types with interaction networks revealed the Epidermal Growth Factor signaling system as a regulator of the extracellular environment [8] and using network analysis a central role of AMPK and energy homeostasis impairment in Alzheimer’s disease was identified [9]. In addition, context-specific networks can be generated based on high-throughput datasets such as transcriptomics [10], proteomics [11] and metabolomics [12]. Data-driven network reconstruction methods, such as Weighted Gene Co-expression Analysis [13] can be used to extract co-regulated network modules that reduce dimensionality and identify biologically relevant patterns in the data*.* These methods have proven to be of value in complex diseases, from defining a network-based inflammation signature common across diseases [14], to elucidating molecular mechanisms underlying autism in brain [15]. To condense useful information out of these large networks, it is necessary to prioritize and extract parts of this network that are most relevant in the context of disease development or response to interventions. Such network signatures aid design of novel, evidence-based therapies by discovering and prioritizing key intervention targets, improving understanding of underlying mechanisms, and can serve as biomarkers for efficacy of interventions [16]-[20].

Current drug treatments to intervene with type 2 diabetes mellitus (T2DM) are considered insufficient [21], and novel interventions are being developed to improve treatment efficacy across the range of T2DM-related complications. A recent preclinical study on anti-diabetic treatments in LDLR-/- mice (ADT study) [22] provides a model to investigate the mechanisms underlying T2DM-associated disease parameters, such as risk factors (e.g. plasma glucose and insulin levels) and complications (e.g. atherosclerosis). This study compared the efficacy of ten anti-diabetic drugs and dietary life style intervention (DLI) on the course of the disease by extensive histological, molecular and biochemical phenotyping. This showed that DLI had a supreme successful effect, reverting nearly all T2DM risk factors and complications to the healthy level. Most drug interventions reduced hyperglycemia but T2DM-associated complications were not improved or, in case of fenofibrate and T0901317, were even aggravated [22]. This suggests that underlying molecular networks affected by DLI are key to successfully resolving disease phenotype while targeting hepatic transcription factors PPARA (fenofibrate) or LXR (T0901317) influence downstream molecular networks in a suboptimal way leading to both beneficial and undesirable phenotypic effects. With this in mind, we used the hepatic transcriptome dataset associated with these three interventions in the ADT study to infer molecular paths that should be either mimicked or circumvented when designing improved interventions.

Our analysis extracted network signatures that link hepatic effects of anti-diabetic interventions with systemic disease parameters using a combination of data-driven and knowledge-based network analysis. This provides a ranking of nodes in the underlying complex regulatory network whose transcriptional regulation is most strongly linked to specific disease parameters. The network signatures allow mechanistic insights into hepatic processes that drive dyslipidemia-associated T2DM phenotypes, place selected genes in functional context of different intervention effects and highlight them as potential novel drug targets.

## 2 Results

**Figure 1:**
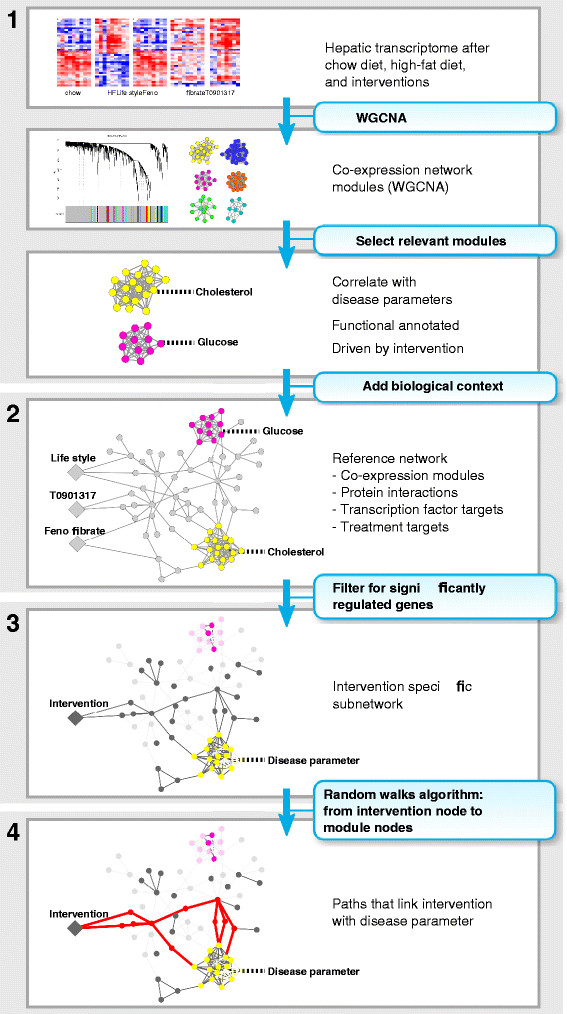
**Schematic representation of the network analysis approach to link intervention targets to disease parameters**. The analysis consists of the following steps: **1)** The hepatic transcriptome dataset is used to build a weighted co-expression network from which modules are selected that are relevant for linking intervention effects to disease parameters. **2)** A reference network is generated by adding knowledge-based biological context to the co-expression modules, including protein interactions, transcription factor targets and intervention targets. **3)** For each intervention, an intervention-specific network is generated by filtering for genes significantly regulated by the intervention. **4)** The kWalks algorithm is applied to the intervention-specific network for each intervention, to extract the most relevant paths linking intervention targets and co-expression modules that link to disease parameters.

To identify network signatures that define transcriptionally regulated paths linking anti-diabetic intervention targets with changes in type 2 diabetes mellitus (T2DM) disease parameters, we applied network analysis on the ADT study [22] which includes both extensive phenotyping of systemic disease parameters as well as hepatic transcriptome measurements. We included 16 measured disease parameters consisting of parameters relevant for insulin resistance (plasma glucose and insulin, QUICKI index), body and organ weights (adipose depots, kidney, liver, heart, and total body weight), atherosclerotic lesion area, plasma cholesterol, and plasma and liver triglycerides. Five experimental groups from the ADT study have been included in our analysis: the chow and high-fat diet (HFD) groups, serving as reference for health and disease state, the dietary lifestyle intervention (DLI) representing successful intervention, and two drug intervention groups (fenofibrate and T0901317, targeting hepatic transcription factors PPARA and LXR, respectively). The following network analysis has been applied to the ADT dataset (Figure 1): 1) Co-expression modules identification and selection, 2) Extension of co-expression modules with different knowledge-based networks to provide a wider biological context and to include intervention targets, 3) Creation of intervention-specific networks by filtering the reference network for differentially expressed genes (DEGs) and 4) extraction of the most relevant paths linking intervention targets with disease parameters. The following sections describe the results of each step.

### 2.1 Identification of co-expression modules

To identify clusters of genes which are co-expressed genes in the context of the interventions, co-expression modules were identified using weighted gene co-expression network analysis (WGCNA) on hepatic gene expression data from the chow, HFD, DLI, fenofibrate, and T0901317 groups. After topological clustering of the co-expression network, 24 modules were identified, varying from 22 to 451 genes (Supplementary Figure S1 in Additional file 1: Table S1 in Additional file 2). For each module an eigengene was calculated that provides a single representation of the profiles of all genes within the module. For 14 modules a valid eigengene could be calculated, that explained minimal 50% of the variance in first principal component (Table 1). Next, identified modules were assessed for their relevance based on whether they could be annotated to a biological process, whether they correlate to one or more disease parameters, and whether they are driven by the interventions. The detailed description of these three analysis steps is provided in Additional file 3.

**Table 1:** Identified network modules

**Module**	**Size**	**GO Terms**	**Significant correlations**
A (yellow)	198	metabolic process; amine metabolic process; negative regulation of peptidase activity; lipid biosynthetic process; oxidoreductase activity; electron carrier activity	Liver weight (-0.91), Triglycerides (-0.90), Atherosclerosis (-0.79), Cholesterol (-0.79)
B (red)	161	cell activation; response to stress; immune system process; inflammatory response; cytokine production	Atherosclerosis (0.80), Cholesterol (0.78), Liver weight (0.75)
C (black)	142	lipid metabolic process; carboxylic acid metabolic process; acyl-CoA metabolic process; thioester metabolic process; oxidation-reduction process	Liver weight (0.88), Cholesterol (0.83)
D (green)	185	aromatic amino acid family catabolic process; cofactor binding; rRNA binding; endopeptidase inhibitor activity	
E (royalblue)	51		Cholesterol (-0.82)
F (blue)	369	protein localization; protein transport; generation of precursor metabolites and energy	
G (magenta)	125	circadian rhythm	
H (purple)	118	regulation of primary metabolic process	
I (greenyellow)	112	receptor activity	
J (cyan)	83		
K (lightyellow)	54		
L (darkgreen)	41		
M (darkturquoise)	39	electron transport chain	
N (orange)	22	hexosaminidase activity	

Modules with valid eigengenes and their size, representative GO terms, and significant correlations with disease parameters (correlation coefficient in parentheses).

Shortly, of the 14 co-expression modules, modules A, B, and C satisfy all three criteria. The module for which the most significantly enriched GO terms were identified was module B, annotated to immune and inflammation related functions. Genes from module A and C were enriched for metabolic process related GO terms. Multiple co-expression modules correlated with the disease parameters related to dyslipidemia, which were previously found to be deteriorated after both drug interventions (plasma and intrahepatic triglycerides, cholesterol, atherosclerotic lesion area, liver weight). In contrast, although glycemia/insulin sensitivity related disease parameters (glucose, insulin, QUICKI) and obesity (body weight, epididymal fat weight) are fully resolved by T0901317 and partly by fenofibrate [22], no co-expression module correlated with these parameters. This indicates that hepatic target activation determines changes in dyslipidemia rather than dysglycemia. The three modules with significant GO annotation and correlation to a disease parameter (A, B, C) are also enriched for genes differentially expressed after HFD, which are largely reversed by DLI. This is in concordance with the observed improvement by DLI of the disease parameters correlating with these modules. In contrast, the drug interventions further deregulate nearly all genes in the module that were also regulated by HFD. These opposite effects match the observed deterioration of the corresponding disease parameters by both drug interventions. In addition, the modules show a large part of additional genes regulated by drugs that were not deregulated by disease, indicating that the drug interventions target or result in different metabolic and immune-related mechanisms than those related to disease progression. Module B, annotated to immune response and inflammation related processes, was most strongly enriched with DEGs for the T0901317 intervention. Notably, the majority of DEGs (141 out of 146) in the module were upregulated by T0901317 compared to HFD and show the opposite response for DLI where these are downregulated compared to HFD. These genes include genes encoding for several macrophage markers (CD14, CD68, LYZ), and immune cell specific proteins (CD86, CD74, CD83, CD52, CD53, Rac2).

### 2.2 Extending co-expression modules with knowledge-based interactions and intervention targets

Modules A, B, and C which are annotated to a biological process, correlated to a disease parameter, and enriched with DEGs in response to intervention were extended with a knowledge-based interaction network and intervention targets. This knowledge-based network comprises different types of interactions, including curated interactions and reactions from pathways databases, experimentally determined protein-protein interactions, and predicted functional protein associations.

The main intervention target for fenofibrate is Peroxisome proliferator-activated receptor alpha (PPARA), which upon activation increases the catabolism of triglycerides by induction of lipoprotein lipase (LPL) and reducing the production of very-low-density lipoprotein (VLDL) [23]. The main targets of T0901317 are Liver X receptor alpha (LXRA) and beta (LXRB), whose activation results in efflux of cholesterol from macrophages in atherosclerotic lesions, which are converted by the liver into bile acids, thereby reducing vascular inflammation and increasing plasma HDL cholesterol [24]. Based on information from the STITCH database [25], 9 additional targets for fenofibrate, and 4 additional targets for T0901317 were included, which comprise secondary and indirect targets as well. For DLI, 21 empirically identified transcription factors whose target genes are enriched among DEGs in the DLI group were included as intervention targets. Together, merging of the three most relevant modules, the knowledge-based interaction network, and the intervention targets resulted in a reference network of 11,970 nodes and 118,493 edges on which further analysis was based.

### 2.3 Filtering reference network for the intervention-specific transcriptional response

For each intervention, a subnetwork was generated based on all genes in the reference network that were differentially expressed under that intervention (DEGs, p < 0.05,). In each case, about 40% of the total number of DEGs is represented in the intervention network and connected with at least one edge (connection between two proteins, representing a functional association or interaction) (Table 2). To investigate the relevance and translational value of the intervention networks we overlaid information derived from human genetic associations relevant to metabolic syndrome and related diseases (obesity, insulin resistance, and type 2 diabetes mellitus) on the intervention networks. A significant enrichment with disease-associated genes was found for each intervention network compared to the total set of measured genes (Fisher’s exact test, DLI: p < 1.33E-15, fenofibrate: 4.86E-26, T0901317: 7.79E-23). This indicates that the dataset and resulting intervention networks indeed capture relevant processes linked to human disease and supports the use of the LDLR-/- mice as model for studying metabolic syndrome in humans.

**Table 2:** Number of DEGs in the dataset and sizes of the intervention-specific networks

	**Total DEGs in dataset (p < 0.05)**	**Connected nodes**	**Edges**
**DLI network**	1287	497	5975
**Fenofibrate network**	2149	828	21598
**T0901317 network**	2924	1245	38472

### 2.4 Network signatures linking intervention to disease parameters

**Figure 2:**
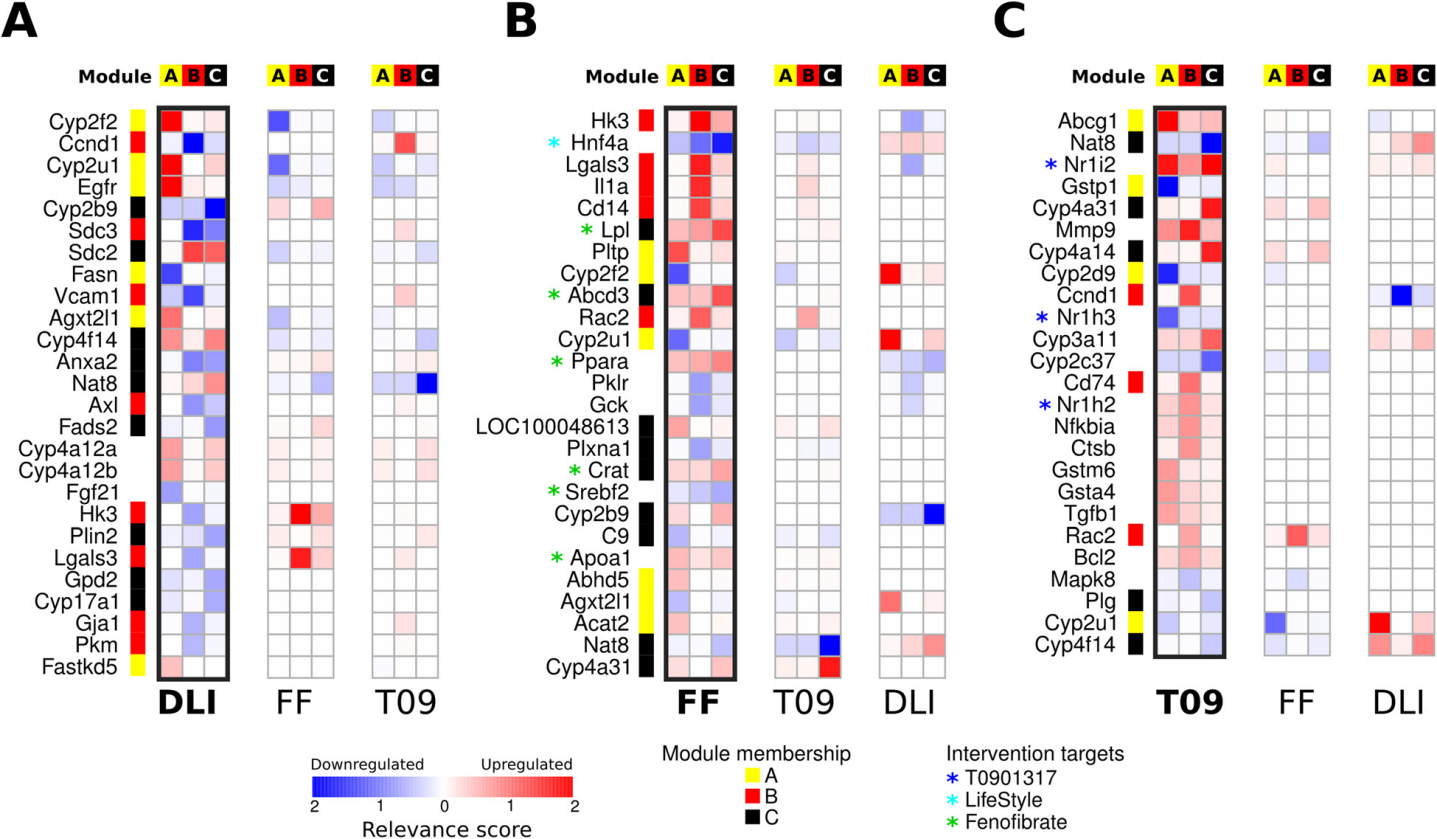
**Identified network signatures**. Network signatures of the top relevance scores representing the centrality of the node in linking the three interventions with disease parameters through co-expression modules **A**, **B**, and **C**. **A)** The signatures for DLI (left bar) and corresponding relevance scores for fenofibrate (FF) and T0901317 (T09) (right bars). **B)** The signatures for fenofibrate (FF, left bar) and corresponding relevance scores for DLI and T0901317 (T09) (right bars). **C)** The signatures for T0901317 (T09, left bar) and corresponding relevance scores for DLI and fenofibrate (FF) (right bars). Each signature contains the union of the top 10 genes with highest relevance scores for connecting the intervention targets to genes from either module **A**, **B**, or **C**. Each column in the heatmap represents the relevance scores for the paths to a module, each row represents a gene in the network. The genes are sorted by the maximum of the relevance scores across the signatures for module **A**, **B**, and **C**. Cells are shaded by relevance score (darker is a higher relevance score) and colored by direction of regulation by the intervention (red is upregulated, blue is downregulated). Colored boxes left of the gene symbols indicate for each gene the co-expression module membership. Colored asterisks on the right of the gene symbols indicate when the gene was considered as intervention target).

For each of the three selected co-expression modules we identified the most relevant paths between intervention targets and any of the module nodes in the corresponding intervention network using the kWalks algorithm. This resulted in a relevance score for each node and edge, representing the expected number of times it is visited by random walks between the intervention and module nodes. These scores provide a ranked network signature for each intervention, highlighting the genes that have the most relevant position in the network in connecting DLI, fenofibrate and T0901317 interventions with co-expression module genes associated to disease parameters atherosclerosis, plasma cholesterol levels, liver weight, and plasma triglyceride levels (Figure 2).

Figure 2A shows the network signature for DLI, composed of the genes with highest module scores for at least one of the three modules, colored by direction of regulation. Many genes have a high relevance score for a single module, but low score for other modules (Cyp2f2, Ccnd1, Cyp2u1, Egfr, Fasn, Agxt2l1, Fads2, Fgf21, Plin2, Fastkd5), indicating that they may serve as good drug targets for specifically affecting this particular disease parameter. Several genes have a high relevance score for multiple modules, such as Sdc3, Sdc2, Anxa2 for the DLI signatures for modules B and C. This may indicate a role for the proteins encoded by these genes in cross-talk between the processes underlying these modules, in this case between lipid metabolism (module C) and inflammation (module B). To compare network signature of DLI to drug interventions, the relevance scores for the drug interventions are also shown in Figure 2A. Most genes with high relevance score for DLI have zero or very low score for the drug interventions (e.g. Fasn, Axl, Fgf21, Gpd2, Cyp17a1, Pkm, Fastkd5, etc.) indicating they are part of DLI-specific mechanisms linked to the positive effect of this intervention. Only 6 out of 26 genes in the DLI signature are also among the genes with top relevance scores for the drug interventions. Notably, these are all regulated in the opposite direction compared to DLI signature. In the DLI profile only Cyp4a12a and Cyp4a12b are regulated in the same direction for both DLI and drugs and have a relevance score for the drugs, albeit low. In addition, none of the proteins encoded by the genes in the DLI signature are targets of either of the two drugs. These observations indicate that the hepatic mechanisms through which the drug interventions aim to resolve disease have little overlap with mechanisms underlying the successful DLI intervention.

Figure 2B and C show the network signatures for the genes with highest relevance scores for the drug interventions. As expected based on the different mechanisms of action, the relevance scores for two drugs show a distinct pattern. A large part of the signature for fenofibrate consists of genes encoding proteins with a role in lipid metabolism and transport, such Lpl, Pltp, Abcd3, Ppara, Crat, Apoa1, Abhd5, Acat2. These genes are upregulated after fenofibrate intervention, corresponding to its known effect on increasing lipolysis through activation of PPARA, resulting in improvement of lipidaemia-related disease parameters (LDL cholesterol, triglycerides) [26]. The signature of T0901317 includes several of its targets as most relevant nodes (Nr1i2, Nr1h3, Nr1h2), as well as immune and inflammation related genes relevant for the module B (Mmp9, Cd74, Nfkbia, Rac2), lipid metabolism relevant for module C (Cyp4a31, Cyp4a14), detoxification and drug-metabolizing proteins relevant for modules A or C (Gstp1, Cyp2d9, Cyp3a11, Cyp2c37). The biological annotation of identified nodes suggests their central role in mediating mechanisms underlying T0901317 effects on disease endpoints, in particular effect on levels of triglyceride and cholesterol in serum and the effect on modulation of inflammatory signals relevant to development of atherosclerosis.

### 2.5 Performance assessment of network-based ranking

**Figure 3:**
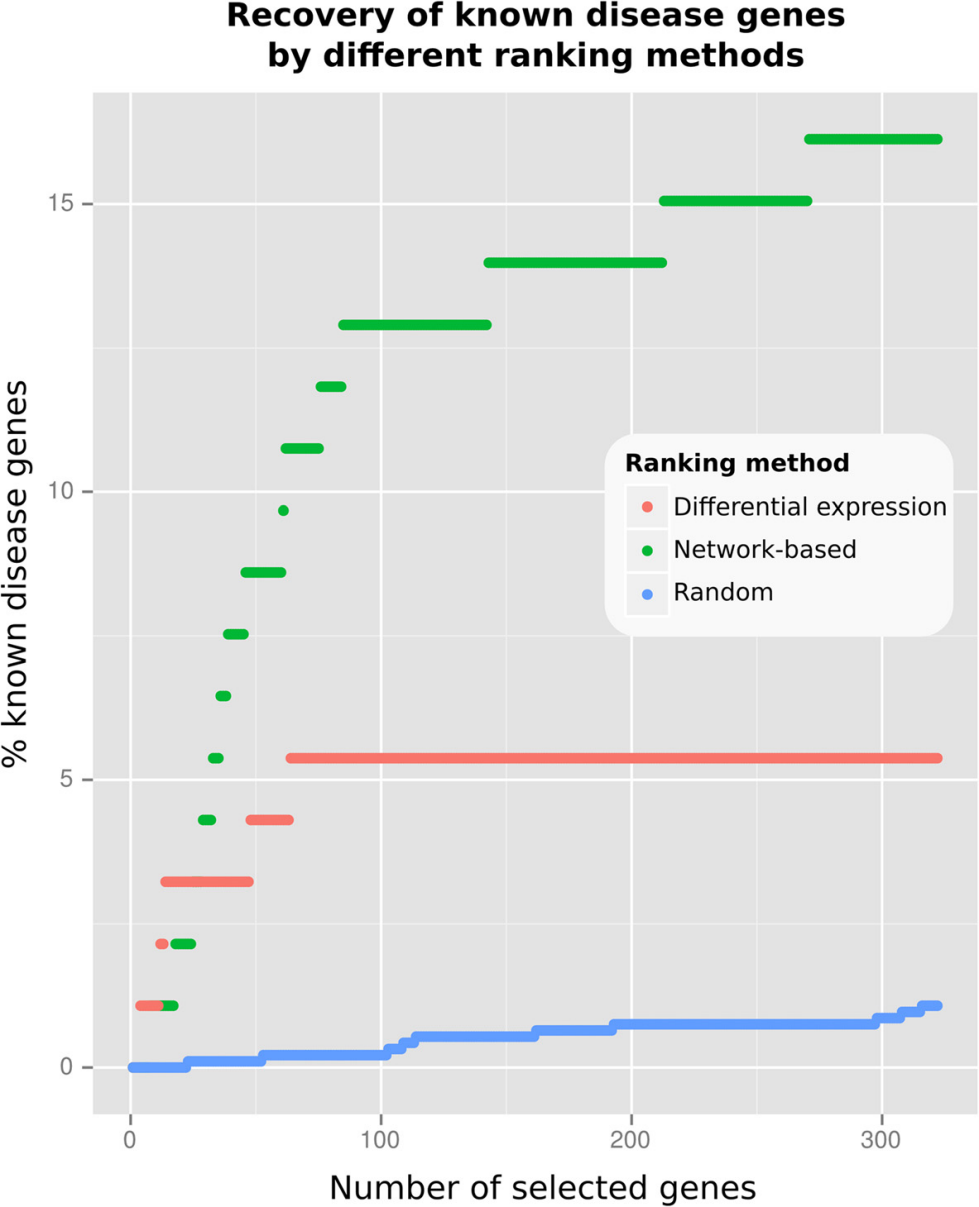
**Comparison of different ranking methods.** Comparison of different ranking methods for the dietary lifestyle intervention (DLI) with respect to the ability to recover genes known to be relevant to hepatic T2DM processes. Plotted are our network-based ranking (green points), ranking by differential expression alone (red points) and random ranking (blue points). The horizontal axis represents the number of selected top genes based on the ranking method, the vertical axis shows the percentage of known disease genes that is present in the set of selected top genes.

To assess whether the gene ranking within network signatures improves our ability to identify top-relevant genes for hepatic processes underlying type 2 diabetes mellitus (T2DM), we compared our network-based ranking with gene ranking by differential expression (p-value) alone and with random ranking. As reference of known disease genes, a list of 93 mouse genes and gene products related to hepatic T2DM processes was generated using text mining of the PubMed database (see Methods). Figure 3 shows the coverage of known disease genes in sets of top ranked genes of increasing size for the ranking based on DLI. As expected, both network-based ranking and ranking based on differential expression outperform random ranking. For sets of 25 genes or more, network-based ranking outperforms ranking based on differential expression by covering more known disease genes, with up to three times higher coverage. In addition, after selecting the top 64 genes, ranking based on differential expression fails to identify any additional known disease genes, while the coverage for the network-based ranking continues to increase. Table 3 shows the enrichment of known disease genes (based on Fisher exact test) in the full network signatures (all genes that were assigned a positive score by the random walks algorithm) and signatures of the same size based on ranking by differential expression. For all three interventions, the network signatures show a higher enrichment with known disease genes than the same number of genes selected based on ranking by differential expression, with 2.8 times higher enrichment for the DLI signature.

**Table 3:** Enrichment of known T2DM genes in the network signatures

**Ranking**	**Fold enrichment**	**P-value**
**DLI network-based**	8.78	3.44E-10
**DLI differential expression**	3.09	0.023
**T0901317 network-based**	3.79	5.09E-06
**T0901317 differential expression**	2.27	0.016
**Fenofibrate network-based**	7.37	1.08E-11
**Fenofibrate differential expression**	2.86	0.011

Enrichment of genes known to be relevant to hepatic T2DM processes based on literature in the full network signatures (all genes that were assigned a positive score by the random walks algorithm), and signatures of the same size based on ranking by differential expression alone. Fold enrichment is the increase of ratio between known relevant genes and remaining genes over the signature compared to the same ratio over all genes in the dataset. The p-values were calculated using a Fisher exact test.

To further validate the relevance of the signatures in the context of drug discovery and healthcare applications, we tested whether the signatures were enriched with known drug targets for T2DM, Fatty liver, or Atherosclerosis and genetic associations to these diseases. The network signature based on DLI showed significant enrichment for drug targets (p = 0.014), while the signature of the same size based on ranking by differential expression was not significantly enriched (p = 0.069). The network signatures for Fenofibrate and T0901317 consistently show a higher, albeit not significant, enrichment with drug targets than the corresponding signatures based on differential expression (Supplemental Table S2 in Additional file 4). For genetic associations, both the network signatures and signatures based on differential expression were significantly enriched, but the network signature showed a consistently higher enrichment (Supplemental Table S2 in Additional file 4).

Finally, we tested translatability and tissue-specificity of the signatures by identifying the coverage of genes in several baseline gene expression datasets in both mouse and human (Supplemental Table S3 in Additional file 5). The signature is well covered for all tissues across mouse and human (61% nodes in signature expressed for tissue with lowest coverage), indicating that the signature has relevance beyond the mouse model used in our dataset. As expected based on the origin of our dataset, liver has best coverage for each signature compared to the other mouse tissues.

### 2.6 Biological context of network signatures

**Figure 4:**
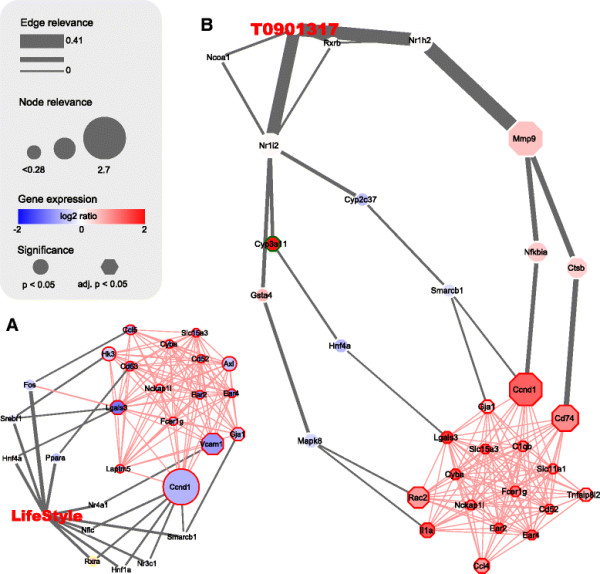
**Network visualization of a subnetwork underlying the signature for module B.** Network visualization of the subnetwork with highest relevance scores for linking intervention targets and module B for DLI **(A)** and T0901317 **(B)**. The subnetwork was obtained by including edges with relevance score above a dynamically obtained cutoff, which is 0.024 for DLI and 0.021 for T0901317, and all edges that connect nodes within module B (pink edges). Nodes with a red border are part of the co-expression module B. Node size and edge weight are scaled by relevance scores as calculated by the kWalks algorithm. Nodes are colored by log2 ratio of intervention versus high-fat diet gene expression and their shape indicates level of significance of differential expression (p < 0.05 has a round shape, FDR adjusted p < 0.05 has an octagon shape).

In addition to identifying a panel of prioritized nodes, the network signatures add a biological context to this ranking by providing information on the relevance of underlying interactions between these nodes. These interactions shed a light on biological pathways that link the signature genes to disease parameters and provide mechanistic insight into interventions effects. To facilitate functional interpretation, for each signature a subnetwork containing the top most relevant interactions and module genes was extracted and visualized (Figure 4, Supplementary Figure S2 and S3 in Additional files 6 and 7).

Figure 4 shows the subnetwork associated to module B, which is strongly enriched with immune and inflammation genes and DEGs for both DLI and T0901317 intervention. This module also shows a clear opposite pattern of regulation between these interventions where the majority of genes were downregulated by DLI, while upregulated by T0901317 intervention. Several nodes receive a non-zero relevance score for both interventions (Ccnd1, Lgals3, Gja1) while the network visualization provides insight in difference in their regulation by the interventions. For example, Ccnd1 has a high relevance score in both signatures, but is downregulated by DLI and upregulated by T0901317. In the DLI network, Ccnd1 is directly regulated by 5 transcription factors affected by DLI, of which 4 could be related to inflammation or immune response pathways (Nr3c1, Nr4a1, Rxra, Smarcb1; based on annotations in Gene Ontology, Ingenuity Pathway Analysis, and WikiPathways). In contrast, Ccnd1 is connected to T0901317 through a single indirect association involving multiple intermediate interactions. This difference can be observed throughout the network, as the average shortest path length from intervention to the module nodes is twice as long in the T0901317 subnetwork compared to the DLI subnetwork. In addition, the edge relevance scores for the DLI network are more equally distributed across nodes, while the scores in the T090137 network are mainly concentrated in the path through Mmp9. This may indicate a more direct and balanced activation of repression of a combination of multiple transcription factors by DLI, while the indirect regulation by T0901317 intervention leads to a less controlled mechanism.

## 3 Discussion

Treatment of complex diseases requires control of the underlying network of molecular processes that translates in a diverse range of complications at the physiological level. For example, in type 2 diabetes mellitus (T2DM) it is not sufficient to solely improve hyperglycemia, as this does not significantly reduce risk for cardiovascular complications [27]. Therefore, it is crucial to study disease in light of the full complexity of the underlying network of interconnected mechanisms contributing to pathology [1]. Here we propose a network-based solution to find hepatic molecular signatures that play a key role in the effect of interventions on different pathologies. By combining a series of network analysis approaches on a hepatic transcriptomics dataset from the preclinical study in an LDLr-/- mouse model fed high fat diet, we discovered and functionally annotated gene co-expression network modules associated with anti-diabetic interventions. We then extracted network signatures representing most critical molecular paths that link the interventions with dyslipidemia-related parameters. The resulting network signatures highlight a panel of ranked-by-relevance, functionally related nodes that may be used either as intervention targets or as biomarkers for determining likelihood of success for novel anti-diabetic interventions.

We integrated different interactions resources, including protein-protein interactions, canonical pathways, and transcription factor targets, to embed the co-expression modules in a larger reference network. This combination of data-driven co-expression networks and knowledge-based functional interactions allowed us to make optimal use of the specific strengths of both approaches: analyze molecular data within the framework of available knowledge to interpret the patterns identified in the data (knowledge-based), but still allowing inclusion of novel interactions not described before (data-driven). By filtering the resulting networks for genes affected by intervention, we were able to extract context-specific networks that were highly enriched for genes genetically linked to T2DM. We exploited the underlying topology of these networks using a subgraph extraction method to further prioritize interactions in the network based on their relevance in connecting known intervention targets with the co-expression modules. Together, our network biology approach provides both a high-level view on patterns in the data by identifying network modules and zooms in to the molecular level by prioritizing the most relevant nodes and interactions to identify putative targets that may aid improved therapy development.

Currently, the network analysis as described here makes use of undirected networks, limiting the analysis to identification of associations rather than cascades of regulatory events that lead to changes in disease parameters. Firstly, the co-expression network analysis identifies associations rather than causal links between molecular changes and disease parameters, so it cannot be determined whether the identified modules are directly driven by intervention or an indirect association. To overcome this problem, causal networks could be derived from studies combining genetic variation with gene expression [18], however to our knowledge no such studies are available in context of anti-diabetic interventions. Secondly, in this workflow different types of interactions are combined, some of which result from high-throughput experiments (protein-protein interactions) or prediction algorithms, which may be less specific and lack directionality. Nevertheless, we chose to include these to provide sufficient coverage, since limiting on a single interaction resource or including only curated and directed interactions would drastically reduce the network size and introduce bias to existing knowledge rather than discovery of novel findings. With current initiatives in pathway and network curation [3],[4],[28] the availability of directed interactions is expected to grow. Since the random walks algorithm we used here can incorporate network directionality, it will be straightforward to include this information in future analyses when sufficient coverage can be obtained with directed networks. Furthermore, when studies measuring tissue-specific regulatory elements [29] and context-specific interactions [30] become abundant, this analysis can be further improved by using this interaction information directly, rather than inferring edge specificity through the connecting nodes.

Despite the evident improvement of glycemic status by all three interventions [22], we found a strong correlation of hepatic transcriptional mechanisms only with dyslipidemia related disease parameters, but not with dysglycemia. This suggests that hepatic mechanisms predominantly determine dyslipidemic phenotypes, as hepatic target activation is rather associated with dyslipidemia than with dysglycemia related disease parameters. The observed improvement in dysglycemia may instead be controlled by organs other than liver. For example, fenofibrate has been shown to influence insulin sensitivity in muscle [31] and adipose tissue [32], and T0901317 can modulate insulin secretion by pancreatic beta cells [33]. This emphasizes the importance of a systems-level understanding of interventions, which requires mapping molecular networks across multiple organs instead of focusing on the apparent target organ of the intervention. The co-expression modules were functionally annotated to processes related to lipid metabolism, and inflammation and immune response, revealing that these hepatic processes are strongly linked to systemic effects on dyslipidemia in the studied interventions. In particular, one module showed a notably high enrichment in immune and inflammation related transcripts. The presence of upregulated macrophage markers and genes expressed specifically in immune cells indicated that this module may represent mRNA measurements of macrophage origin. We found that this module exhibits a distinct pattern between interventions, where nearly all genes were downregulated by DLI but upregulated by T0901317 intervention. This provides a key hepatic signature linked to improvement or detoriation of dyslipidemia related parameters by DLI and T0901317 intervention.

The network signatures resulting from this analysis provide ranking scores for genes based on their relevance in mediating the effect that each intervention has on disease parameters. By using a random walks based algorithm we were able to encode the structure of the underlying transcriptionally regulated networks in this ranking. The importance of each node is scored according to the centrality of its position along the paths connecting intervention targets with the co-expression modules. We showed that this network-based approach outperforms ranking by differential expression alone. Based on these signatures, we propose genes which have a key role in linking interventions and disease parameter. Genes with a high relevance score unique in the DLI signature, but not for the drug interventions (Fasn, Axl, Fgf21, Gpd2, Cyp17a1, Pkm, Fastkd5), may point to putative targets for improved interventions mimicking the mechanisms underlying DLI. Notably, the gene products of two of these genes are already under investigation as therapeutic targets. Fgf21, encoding for Fibroblast growth factor 21, is currently being investigated as novel therapeutic agent for T2DM [34],[35], and the anti-diabetic properties of the fatty acid synthase (Fasn) inhibitor platensimycin have recently been demonstrated in a mouse model [36]. Interestingly, Axl, encoding for the AXL receptor tyrosine kinase, was found to induce T2DM after overexpression in transgenic mice [37]. In addition, we highlighted a possible key role in cross-talk between inflammatory and metabolic processes (Sdc2, Sdc3, Anxa2) in context of the DLI intervention. Both Sdc2 and Sdc3 encode for syndecans which function as co-receptors in various growth factors signaling pathways and play a role in inflammation as well as lipid metabolism [38]. The therapeutic relevance of this finding may reside in their potential use in uncoupling the inflammation component from metabolic dysfunction which is known to play a critical role in T2DM [39]. In addition to a network based ranking of genes, the network signatures provide visualization that reveal the underlying interactions among the top ranked nodes. This facilitates interpreting mechanisms and substantiating evidence by putting the genes in context of existing knowledge underlying each interaction. Such integration of resources allows for optimal utilization of ever-growing body of publicly available knowledge and data to address research problems of specific interest.

## 4 Conclusions

In summary, by combining data- and prior knowledge-driven networks, correlation analysis, functional annotation and random walks based network analysis, we identified relevance-ranked hepatic network signatures underlying effects of different anti-diabetic interventions on dyslipidemia-related disease parameters. We propose that the DLI network signature may serve as a template for defining new or better intervention targets that mediate global positive effects of this intervention. In turn, network signatures of the two drugs assist in further specification of optimal intervention targets, as these possibly correlate to adverse effects and as such should be avoided.

## 5 Methods

### 5.1 Transcriptomics dataset

The generation of the transcriptomics dataset of the ADT study used in this analysis has been described in [22]. The dataset was obtained from the ArrayExpress database (E-MTAB-1063) [40]. This dataset consists of hepatic transcriptomics measurements with the Illumina MouseRef-8 v2.0 Expression BeadChip platform on LDLr-/- mice that were fed either chow diet or high-fat diet (HFD) for 16 weeks. At 9 weeks, mice from the HFD group were either kept on HFD, switched back to chow diet (dietary life style intervention, DLI), or treated with a drug intervention on top of the HFD (the groups for T0901317 and fenofibrate were included in this analysis). The data was normalized using quantile normalization as described in [22]. Differential expression p-values between the HFD group at 16 weeks (HF16wk) and each intervention group (dietary life style intervention (DLI), Fenofibrate, T0901317) were calculated using the limma R package [41]. Adjusted p-values were calculated using the Benjamini-Hochberg method. For filtering the networks unadjusted p-values were used, whereas for discussing individual genes in the text we used the adjusted p-value.

### 5.2 Knowledge-based network

The knowledge-based network was generated by integrating relations between genes/proteins from different resources, including curated pathways (WikiPathways v20120408 [4], KEGG v20110518 [42], and a set of internally curated pathways), protein interactions (STRING v9 [43], including all interactions with score >0.4), transcription factor targets (Tfe [44] v20111018, Ingenuity Pathway Analysis), known drug-protein interactions for Fenofibrate and T0901317 (STITCH 3.1 [25]). The knowledge-based networks used as input are available in the GML format (Additional file 8).

### 5.3 Co-expression network modules

Gene co-expression modules were identified using the WGCNA package for R [13]. Pairwise gene correlations were calculated based on the expression profile over the hepatic samples from the ADT study at timepoint 16 weeks, in the chow diet, HFD, DLI, Fenofibrate, and T0901317 treated groups. A soft threshold function (power = 4) was applied to the resulting adjacency matrix based on the criterion of approximate scale-free topology (minimum power for which the scale-free fit coefficient > 0.9). The resulting network was clustered into modules by performing hierarchical clustering on the topological overlap matrix (TOM) and using the TreeCut algorithm to define modules (hybrid mode without PAM stage, minimal module size of 20 genes) [45]. Each module was annotated to Gene Ontology using the Bioconductor package GOstats [46], using the hypogeometric test.

The knowledge-based network and the nodes and edges within the co-expression modules were combined to form a single reference network on which further analysis was applied. This was done by taking the union of nodes and edges of both networks followed by merging any multiple edges into single edges.

### 5.4 Correlation between co-expression modules and disease parameters

The ADT study [22] included measurements on 16 disease parameters. These include plasma glucose and insulin, QUICKI index, body and organ weights (adipose depots, kidney, liver, heart, and total body weight), atherosclerotic lesion area, plasma cholesterol, and plasma and liver triglycerides (Additional file 9). To associate the co-expression network modules to changes in these disease parameters, the eigengene (first principal component, see [22]) of each module was correlated to the measured physiological parameters by Pearson correlation. To ensure the eigengene sufficiently represented the expression profiles of the module genes, modules for which the first principle component explained less than 50% of the variation were excluded from the network.

### 5.5 Intervention-specific networks

To make the reference network intervention-specific, a subnetwork was generated for each intervention by filtering the reference network for corresponding differentially expressed genes relative to the HF diet group. Three intervention-specific subnetworks were generated (the DLI network, Fenofibrate network and T0901317 network), by filtering all differentially expressed genes using a moderate significance threshold (unadjusted p < 0.05). To allow for modeling the drug response, nodes that represent the drug or direct drug targets were included regardless of their differential expression. For the DLI network, a virtual “DLI” node was added and connected to the transcription factors of which the target sets were significantly enriched with differentially expressed genes (overlap P value < 0.001, Ingenuity Pathway Analysis [47]).

### 5.6 Disease associations

To assess the translational relevance of the response networks, information on human genetic disease associations was combined with the genes in the network. A list of human genes was compiled which were genetically associated with any of a set of metabolic syndrome related phenotypes (T2DM, insulin resistance, metabolic syndrome, obesity) based on the genetic association database (v11102011) [48] and HuGE Navigator Phenopedia (v11102011) [49]. These genes were mapped when possible to their mouse ortholog using mappings provided by Ensembl (version 64) [50].

To assess the ranking performance of the network signatures for genes relevant to hepatic T2DM related processes, we used the text mining tool Fable (http://fable.chop.edu) to create a reference list of known functionally relevant genes or proteins. We applied the PubMed query “‘type 2 diabetes mellitus’ AND liver” in Fable to compile a list of 491 human protein names. As with the genetic associations, the resulting genes were mapped when possible to their mouse ortholog using mappings provided by Ensembl (version 64), resulting in a reference list of 93 mouse genes.

Enrichment with drug targets and genetically associated genes was calculated using the Hypergeometric test. Drug targets were obtained from DrugBank, by querying for targets of drugs acting on T2DM, Fatty liver, or Atherosclerosis. For genetic associations, the set of genes obtained from the genetic association database and HuGE Navigator Phenopedia (see above) was used.

### 5.7 Tissue specificity and translatability

Genes in the signatures were cross-referenced with RNA-Seq baseline experiments from ArrayExpress Atlas [51], providing information on whether the gene is expressed under baseline conditions in 6 mouse tissues and 27 human tissues (E-MTAB-599 and E-MTAB-1733). The default expression level cutoff of 0.5 FPKM (Fragments Per Kilobase of transcript per Million mapped reads) was used. Human genes were mapped when possible to their mouse ortholog using mappings provided by Ensembl (version 64).

### 5.8 Subnetworks linking intervention and disease parameters

To identify subnetworks relevant for the relationship between a drug and a disease parameter, the kWalks algorithm [52] was used. The kWalks algorithm aims to extract a subnetwork that best explains the relationships between a set of given nodes of interest in a network. In this case, the nodes of interest are the node for a given drug, and the nodes in a given co-expression module which correlates to a disease parameter. For each combination of drug and co-expression module, relevance scores were calculated for each node and edge in the corresponding response network, representing the expected number of times the node or edge is visited by random walks between the drug node and module nodes. To increase performance, the maximum path length of the random walker (L_max_) was set to 50, which was shown to approximate the case of using unlimited random walks very well [52].

The resulting node and edge relevances were used to extract the most relevant paths from drug origin to disease parameters. For visualization of the most relevant subnetworks, a cutoff for the edge relevance score was identified that minimizes the complexity of the network, while keeping both the drug and module nodes connected. We identified the maximum edge relevance score (r_max_) for which the drug nodes and a minimum of 5 module nodes are in the same connected component after removing all edges below r_max_. If no such r_max_ could be identified, the minimum number of required module nodes was decreased until a valid r_max_ could be found. To include the core of the co-expression module in the visualization, the resulting network was combined with the top ten module genes with the highest module membership score (correlation with the module's eigengene) and their edges.

### 5.9 Network processing and visualization

The networks were loaded and processed in R using the igraph package [53]. For network visualization, Cytoscape (version 2.8.1) [54] was used. The resulting network visualizations are available in the Cytoscape session file format (Additional file 10).

All R scripts and the required input data used in this analysis are available as a repository on GitHub (https://github.com/thomaskelder/ADT-liver-network).

## Competing interests

The authors declare that they have no competing interests.

## Authors’ contributions

Conceived the study: TK and MR. Performed the data analysis: TK. Interpretation of results: MR, TK, LV, AJvG and BvO. Wrote the manuscript: TK, MR. Extensive reviewing and editing of the manuscript: LV, AJvG, BvO. All authors have read and approved the final manuscript.

## Additional files

## Supplementary Material

Additional file 1: Figure S1. Dendrogram and module assignment (colors in bar below dendrogram) of the topological clustering of the co-expression network. Click here for file

Additional file 2: Table S1. This table contains the module assignments for each gene in the dataset, as well as corresponding module membership scores and p-values (based on correlation to the module eigengene). Click here for file

Additional file 3: **Module selection analysis.** This document contains a detailed description of the analysis on all co-expression modules in order to select those relevant for further analysis. Click here for file

Additional file 4: Table S2. Results of enrichment analysis of the signatures with drug targets and genetically associated genes. Click here for file

Additional file 5: Table S3. Tissue specificity analysis of the signatures. Click here for file

Additional file 6: Figure S2. Network visualization of a subnetwork underlying the signature for module C, see Figure 4 for detailed legend. Click here for file

Additional file 7: Figure S3. Network visualization of a subnetwork underlying the signature for module A, see Figure 4 for detailed legend. Click here for file

Additional file 8: **Knowledge-based networks used as input.** Contains a zip archive with the GML files for each knowledge-based network used in this analysis. The GML format (http://en.wikipedia.org/wiki/Graph_Modelling_Language) can be read using the R igraph library or Cytoscape (http://www.cytoscape.org). Click here for file

Additional file 9: **Disease parameters.** Excel table containing the measurements on the disease parameters from the ADT study. Click here for file

Additional file 10: **Cytoscape network visualizations.** Cytoscape session file containing the network visualizations as displayed in Figure 4, supplementary Figure S3, and supplementary Figure S4. This file format can be opened using Cytoscape (http://www.cytoscape.org) version 2.8. Click here for file

## References

[B1] Barabási A-L Gulbahce N Loscalzo J Network medicine: a network-based approach to human disease Nat Rev Genet 2011 12 56 68 10.1038/nrg2918 21164525 PMC3140052

[B2] Stumpf MPH Thorne T de Silva E Stewart R An HJ Lappe M Wiuf C Estimating the size of the human interactome Proc Natl Acad Sci U S A 2008 105 6959 6964 10.1073/pnas.0708078105 18474861 PMC2383957

[B3] Croft D O’Kelly G Wu G Haw R Gillespie M Matthews L Caudy M Garapati P Gopinath G Jassal B Jupe S Kalatskaya I Mahajan S May B Ndegwa N Schmidt E Shamovsky V Yung C Birney E Hermjakob H D’Eustachio P Stein L Kataskaya I Reactome: a database of reactions, pathways and biological processes Nucleic Acids Res 2010 39 D691 D697 10.1093/nar/gkq1018 21067998 PMC3013646

[B4] Kelder T van Iersel MP Hanspers K Kutmon M Conklin BR Evelo CT Pico AR WikiPathways: building research communities on biological pathways Nucleic Acids Res 2012 40 Database issue D1301 D1307 10.1093/nar/gkr1074 22096230 PMC3245032

[B5] Kelley R Ideker T Systematic interpretation of genetic interactions using protein networks Nat Biotechnol 2005 23 561 566 10.1038/nbt1096 15877074 PMC2814446

[B6] Rual J-F Venkatesan K Hao T Hirozane-Kishikawa T Dricot A Li N Berriz GF Gibbons FD Dreze M Ayivi-Guedehoussou N Klitgord N Simon C Boxem M Milstein S Rosenberg J Goldberg DS Zhang LV Wong SL Franklin G Li S Albala JS Lim J Fraughton C Llamosas E Cevik S Bex C Lamesch P Sikorski RS Vandenhaute J Zoghbi HY Towards a proteome-scale map of the human protein-protein interaction network Nature 2005 437 1173 1178 10.1038/nature04209 16189514

[B7] Gerstein MB Kundaje A Hariharan M Landt SG Yan K-K Cheng C Mu XJ Khurana E Rozowsky J Alexander R Min R Alves P Abyzov A Addleman N Bhardwaj N Boyle AP Cayting P Charos A Chen DZ Cheng Y Clarke D Eastman C Euskirchen G Frietze S Fu Y Gertz J Grubert F Harmanci A Jain P Kasowski M Architecture of the human regulatory network derived from ENCODE data Nature 2012 489 91 100 10.1038/nature11245 22955619 PMC4154057

[B8] Waters KM Liu T Quesenberry RD Willse AR Bandyopadhyay S Kathmann LE Weber TJ Smith RD Wiley HS Thrall BD Network analysis of epidermal growth factor signaling using integrated genomic, proteomic and phosphorylation data PLoS One 2012 7 e34515 10.1371/journal.pone.0034515 22479638 PMC3315547

[B9] Caberlotto L Lauria M Nguyen T-P Scotti M The central role of AMP-kinase and energy homeostasis impairment in Alzheimer’s disease: a multifactor network analysis PLoS One 2013 8 e78919 10.1371/journal.pone.0078919 24265728 PMC3827084

[B10] Metzker ML Sequencing technologies - the next generation Nat Rev Genet 2010 11 31 46 10.1038/nrg2626 19997069

[B11] Mann M Proteomics for biomedicine: a half-completed journey EMBO Mol Med 2012 4 75 77 10.1002/emmm.201100198 22278912 PMC3376839

[B12] Thiele I Swainston N Fleming RMT Hoppe A Sahoo S Aurich MK Haraldsdottir H Mo ML Rolfsson O Stobbe MD Thorleifsson SG Agren R Bölling C Bordel S Chavali AK Dobson P Dunn WB Endler L Hala D Hucka M Hull D Jameson D Jamshidi N Jonsson JJ Juty N Keating S Nookaew I Le Novère N Malys N Mazein A A community-driven global reconstruction of human metabolism Nat Biotechnol 2013 31 5 419 425 10.1038/nbt.2488 23455439 PMC3856361

[B13] Langfelder P Horvath S WGCNA: an R package for weighted correlation network analysis BMC Bioinformatics 2008 9 559 10.1186/1471-2105-9-559 19114008 PMC2631488

[B14] Wang I-M Zhang B Yang X Zhu J Stepaniants S Zhang C Meng Q Peters M He Y Ni C Slipetz D Crackower MA Houshyar H Tan CM Asante-Appiah E O’Neill G Luo MJ Thieringer R Yuan J Chiu C-S Lum PY Lamb J Boie Y Wilkinson HA Schadt EE Dai H Roberts C Systems analysis of eleven rodent disease models reveals an inflammatome signature and key drivers Mol Syst Biol 2012 8 594 10.1038/msb.2012.24 22806142 PMC3421440

[B15] Voineagu I Wang X Johnston P Lowe JK Tian Y Horvath S Mill J Cantor RM Blencowe BJ Geschwind DH Transcriptomic analysis of autistic brain reveals convergent molecular pathology Nature 2011 474 380 384 10.1038/nature10110 21614001 PMC3607626

[B16] Hasan S Bonde BK Buchan NS Hall MD Network analysis has diverse roles in drug discovery Drug Discov Today 2012 00 1 6 10.1016/j.drudis.2012.05.006 22627007

[B17] Brown JB Okuno Y Systems biology and systems chemistry: new directions for drug discovery Chem Biol 2012 19 23 28 10.1016/j.chembiol.2011.12.012 22284351

[B18] Kasarskis A Yang X Schadt E Integrative genomics strategies to elucidate the complexity of drug response Pharmacogenomics 2011 12 1695 1715 10.2217/pgs.11.115 22118053

[B19] Leung A Bader GD Reimand J HyperModules: identifying clinically and phenotypically significant network modules with disease mutations for biomarker discovery Bioinformatics 2014 30 2230 2232 10.1093/bioinformatics/btu172 24713437 PMC4103591

[B20] Hofree M Shen JP Carter H Gross A Ideker T Network-based stratification of tumor mutations Nat Methods 2013 10 1108 1115 10.1038/nmeth.2651 24037242 PMC3866081

[B21] Nolan CJ Damm P Prentki M Type 2 diabetes across generations: from pathophysiology to prevention and management Lancet 2011 378 169 181 10.1016/S0140-6736(11)60614-4 21705072

[B22] Radonjic M Wielinga PY Wopereis S Kelder T Goelela VS Verschuren L Toet K van Duyvenvoorde W van der Werff van der Vat B Stroeve JHM Cnubben N Kooistra T van Ommen B Kleemann R Differential Effects of Drug Interventions and Dietary Lifestyle in Developing Type 2 Diabetes and Complications: A Systems Biology Analysis in LDLr−/−Mice PLoS One 2013 8 e56122 10.1371/journal.pone.0056122 23457508 PMC3574110

[B23] Rosenson RS Fenofibrate: treatment of hyperlipidemia and beyond Expert Rev Cardiovasc Ther 2008 6 1319 1330 10.1586/14779072.6.10.1319 19018684

[B24] Tontonoz P Mangelsdorf DJ Liver X receptor signaling pathways in cardiovascular disease Mol Endocrinol 2003 17 985 993 10.1210/me.2003-0061 12690094

[B25] Kuhn M Szklarczyk D Franceschini A Campillos M von Mering C Jensen LJ Beyer A Bork P STITCH 2: an interaction network database for small molecules and proteins Nucleic Acids Res 2010 38 Database issue D552 D556 10.1093/nar/gkp937 19897548 PMC2808890

[B26] Keating GM Fenofibrate: a review of its lipid-modifying effects in dyslipidemia and its vascular effects in type 2 diabetes mellitus Am J Cardiovasc Drugs 2011 11 227 247 10.2165/11207690-000000000-00000 21675801

[B27] Gerstein HC Miller ME Byington RP Goff DC Bigger JT Buse JB Cushman WC Genuth S Ismail-Beigi F Grimm RH Probstfield JL Simons-Morton DG Friedewald WT Effects of intensive glucose lowering in type 2 diabetes N Engl J Med 2008 358 2545 2559 10.1056/NEJMoa0802743 18539917 PMC4551392

[B28] Turinsky AL Razick S Turner B Donaldson IM Wodak SJ Literature curation of protein interactions: measuring agreement across major public databases Database (Oxford) 2010 2010 baq026 10.1093/database/baq026 21183497 PMC3011985

[B29] Shen Y Yue F McCleary DF Ye Z Edsall L Kuan S Wagner U Dixon J Lee L Lobanenkov VV Ren B A map of the cis-regulatory sequences in the mouse genome Nature 2012 488 7409 116 120 10.1038/nature11243 22763441 PMC4041622

[B30] Ideker T Krogan NJ Differential network biology Mol Syst Biol 2012 8 565 10.1038/msb.2011.99 22252388 PMC3296360

[B31] Furuhashi M Ura N Murakami H Hyakukoku M Yamaguchi K Higashiura K Shimamoto K Fenofibrate improves insulin sensitivity in connection with intramuscular lipid content, muscle fatty acid-binding protein, and beta-oxidation in skeletal muscle J Endocrinol 2002 174 321 329 10.1677/joe.0.1740321 12176671

[B32] Jeong S Yoon M Fenofibrate inhibits adipocyte hypertrophy and insulin resistance by activating adipose PPARalpha in high fat diet-induced obese mice Exp Mol Med 2009 41 397 405 10.3858/emm.2009.41.6.045 19322024 PMC2705860

[B33] Efanov AM Sewing S Bokvist K Gromada J Liver X Receptor Activation Stimulates Insulin Secretion via Modulation of Glucose and Lipid Metabolism in Pancreatic Beta-Cells Diabetes 2004 53 S75 S78 10.2337/diabetes.53.suppl_3.S75 15561926

[B34] Kharitonenkov A Shiyanova TL Koester A Ford AM Micanovic R Galbreath EJ Sandusky GE Hammond LJ Moyers JS Owens RA Gromada J Brozinick JT Hawkins ED Wroblewski VJ Li D-S Mehrbod F Jaskunas SR Shanafelt AB FGF-21 as a novel metabolic regulator J Clin Invest 2005 115 1627 1635 10.1172/JCI23606 15902306 PMC1088017

[B35] Berglund ED Li CY Bina HA Lynes SE Michael MD Shanafelt AB Kharitonenkov A Wasserman DH Fibroblast growth factor 21 controls glycemia via regulation of hepatic glucose flux and insulin sensitivity Endocrinology 2009 150 4084 4093 10.1210/en.2009-0221 19470704 PMC2736088

[B36] Wu M Singh SB Wang J Chung CC Salituro G Karanam BV Lee SH Powles M Ellsworth KP Lassman ME Miller C Myers RW Tota MR Zhang BB Li C Antidiabetic and antisteatotic effects of the selective fatty acid synthase (FAS) inhibitor platensimycin in mouse models of diabetes Proc Natl Acad Sci U S A 2011 108 5378 5383 10.1073/pnas.1002588108 21389266 PMC3069196

[B37] Augustine KA Rossi RM Van G Housman J Stark K Danilenko D Varnum B Medlock E Noninsulin-dependent diabetes mellitus occurs in mice ectopically expressing the human Axl tyrosine kinase receptor J Cell Physiol 1999 181 433 447 10.1002/(SICI)1097-4652(199912)181:3<433::AID-JCP7>3.0.CO;2-Y 10528229

[B38] Götte M Syndecans in inflammation FASEB J 2003 17 575 591 10.1096/fj.02-0739rev 12665470

[B39] Hotamisligil GS Inflammation and metabolic disorders Nature 2006 444 860 867 10.1038/nature05485 17167474

[B40] Brazma A Parkinson H Sarkans U Shojatalab M Vilo J Abeygunawardena N Holloway E Kapushesky M Kemmeren P Lara GG Oezcimen A Rocca-Serra P Sansone S-A ArrayExpress–a public repository for microarray gene expression data at the EBI Nucleic Acids Res 2003 31 68 71 10.1093/nar/gkg091 12519949 PMC165538

[B41] Smyth GK Linear models and empirical bayes methods for assessing differential expression in microarray experiments Stat Appl Genet Mol Biol 2004 3 1 10.2202/1544-6115.1027 16646809

[B42] Kanehisa M Goto S KEGG: Kyoto encyclopedia of genes and genomes Nucleic Acids Res 2000 28 1 27 30 10.1093/nar/28.1.27 10592173 PMC102409

[B43] Von Mering C Jensen LJ Snel B Hooper SD Krupp M Foglierini M Jouffre N Huynen MA Bork P STRING: known and predicted protein-protein associations, integrated and transferred across organisms Nucleic Acids Res 2005 33 Database issue D433 D437 10.1093/nar/gki005 15608232 PMC539959

[B44] Yusuf D Butland SL Swanson MI Bolotin E Ticoll A Cheung WA Zhang XYC Dickman CTD Fulton DL Lim JS Schnabl JM Ramos OHP Vasseur-Cognet M de Leeuw CN Simpson EM Ryffel GU Lam EW-F Kist R Wilson MSC Marco-Ferreres R Brosens JJ Beccari LL Bovolenta P Benayoun BA Monteiro LJ Schwenen HDC Grontved L Wederell E Mandrup S Veitia RA The transcription factor encyclopedia Genome Biol 2012 13 R24 10.1186/gb-2012-13-3-r24 22458515 PMC3439975

[B45] Langfelder P Zhang B Horvath S Defining clusters from a hierarchical cluster tree: the Dynamic Tree Cut package for R Bioinformatics 2008 24 719 720 10.1093/bioinformatics/btm563 18024473

[B46] Falcon S Gentleman R Using GOstats to test gene lists for GO term association Bioinformatics 2007 23 257 258 10.1093/bioinformatics/btl567 17098774

[B47] http://www.ingenuity.com/products/pathways_analysis.html **Ingenuity IPA Software.**.

[B48] Becker KG Barnes KC Bright TJ Wang SA The genetic association database Nat Genet 2004 36 431 432 10.1038/ng0504-431 15118671

[B49] Yu W Gwinn M Clyne M A navigator for human genome epidemiology Nat Genet 2008 40 2 124 125 10.1038/ng0208-124 18227866

[B50] Flicek P Amode MR Barrell D Beal K Brent S Chen Y Clapham P Coates G Fairley S Fitzgerald S Gordon L Hendrix M Hourlier T Johnson N Kähäri A Keefe D Keenan S Kinsella R Kokocinski F Kulesha E Larsson P Longden I McLaren W Overduin B Pritchard B Riat HS Rios D Ritchie GRS Ruffier M Schuster M Ensembl 2011 Nucleic Acids Res 2011 39 Database issue D800 D806 10.1093/nar/gkq1064 21045057 PMC3013672

[B51] Petryszak R Burdett T Fiorelli B Fonseca NA Gonzalez-Porta M Hastings E Huber W Jupp S Keays M Kryvych N McMurry J Marioni JC Malone J Megy K Rustici G Tang AY Taubert J Williams E Mannion O Parkinson HE Brazma A Expression Atlas update–a database of gene and transcript expression from microarray- and sequencing-based functional genomics experiments Nucleic Acids Res 2014 42 Database issue D926 D932 10.1093/nar/gkt1270 24304889 PMC3964963

[B52] Dupont P, Callut J, Dooms G, Monette JN, Deville Y, Sainte BP: **Relevant subgraph extraction from random walks in a graph.** *Res Report RR* 2006, 380167.

[B53] Csárdi G Nepusz T The igraph software package for complex network research InterJournal Complex Syst 2006 1695 1695

[B54] Smoot ME Ono K Ruscheinski J Wang P Ideker T Cytoscape 2.8: new features for data integration and network visualization Bioinformatics 2011 27 431 432 10.1093/bioinformatics/btq675 21149340 PMC3031041

