# Supplementary figures and images for: Network signatures link hepatic effects of anti-diabetic interventions with systemic disease parameters
Source: BMC Syst Biol. 2014 Sep 11;8:108. doi: 10.1186/s12918-014-0108-0 (PMC4363943; doi:10.1186/s12918-014-0108-0)

Cluster Dendrogram

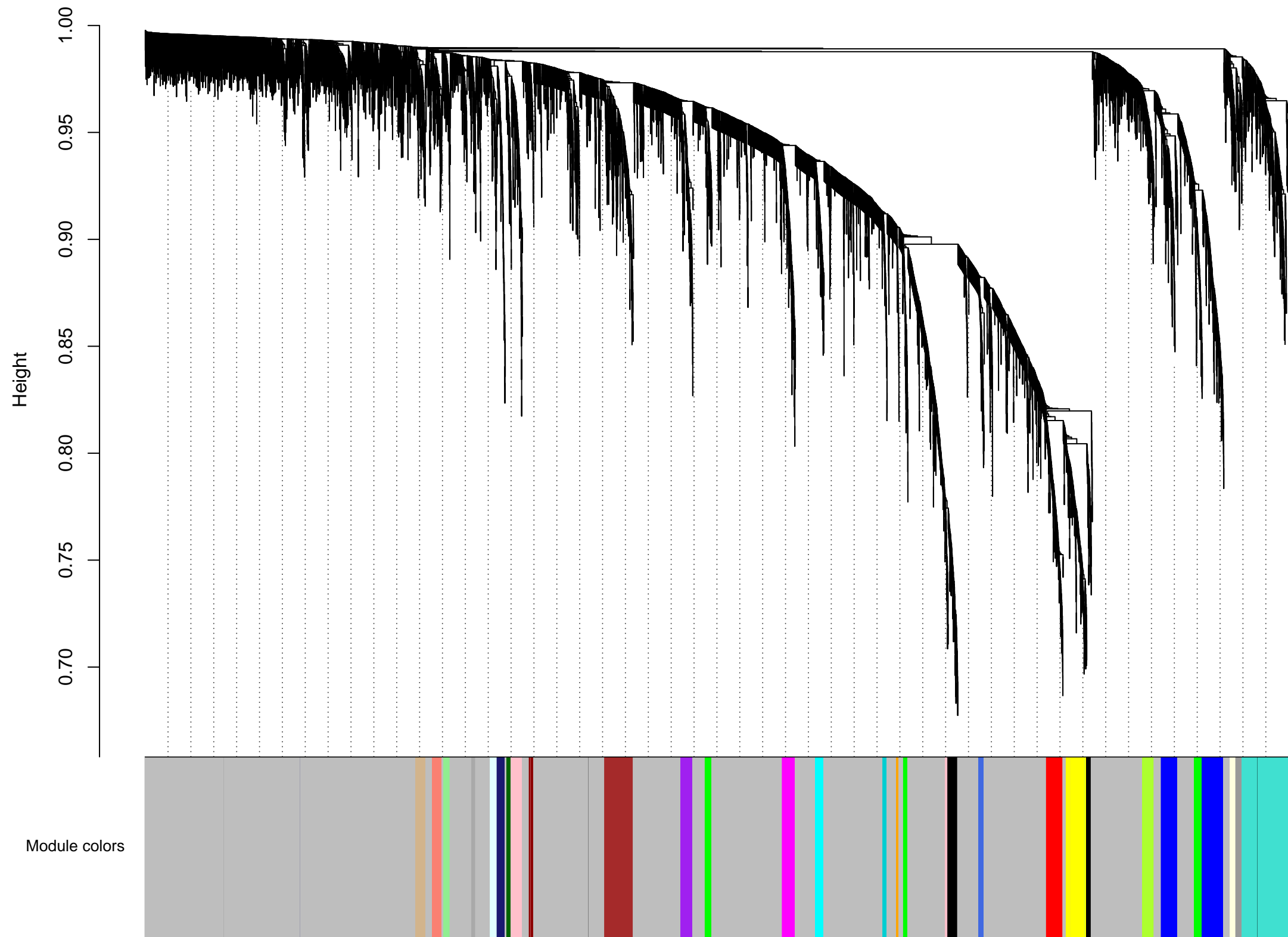

Supplement: Additional file 1: Figure S1. — Dendrogram and module assignment (colors in bar below dendrogram) of the topological clustering of the co-expression network. [file s12918-014-0108-0-S1.pdf]

**B**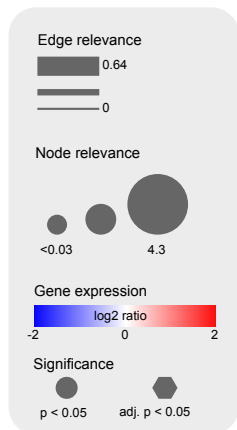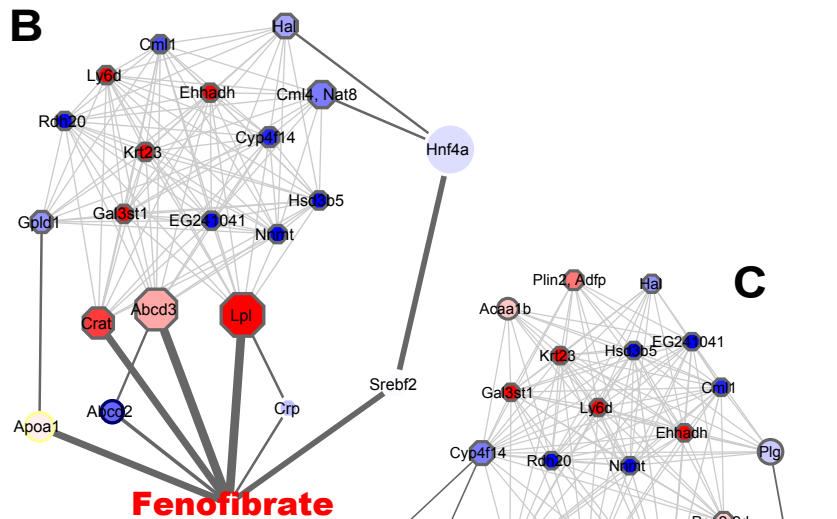**C**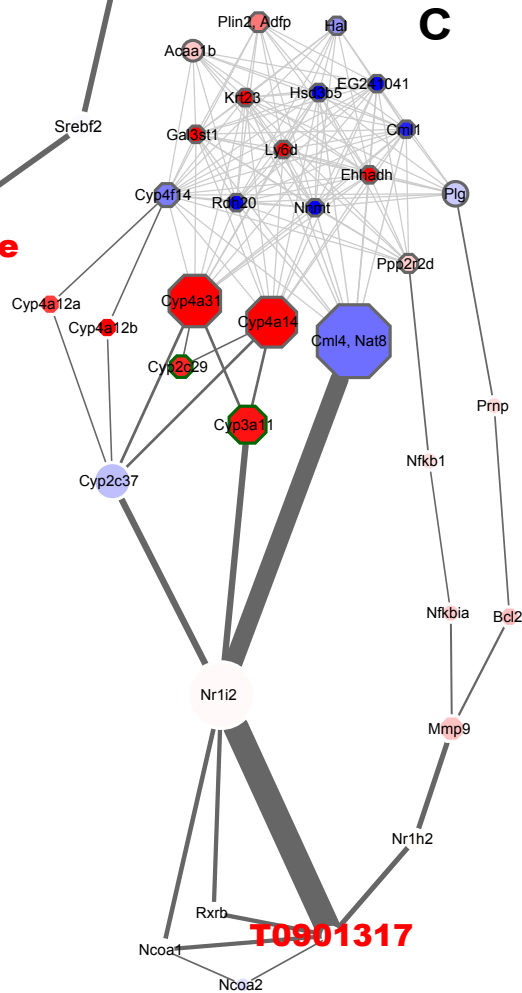**A**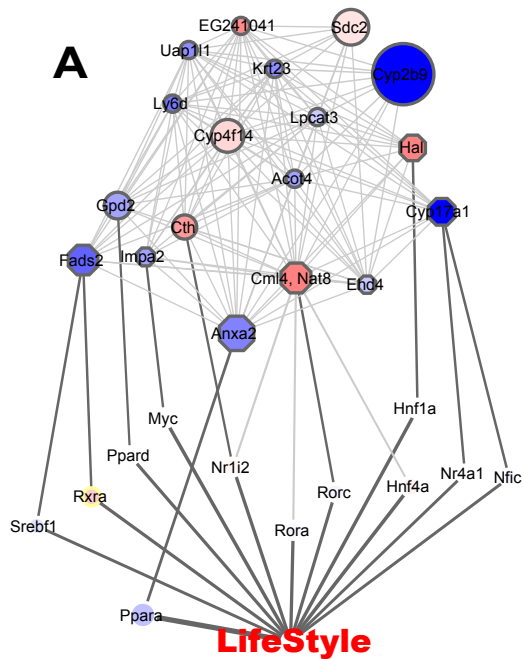

Supplement: Additional file 6: Figure S2. — Network visualization of a subnetwork underlying the signature for module C, see Figure 4 for detailed legend. [file s12918-014-0108-0-S6.pdf]

**A**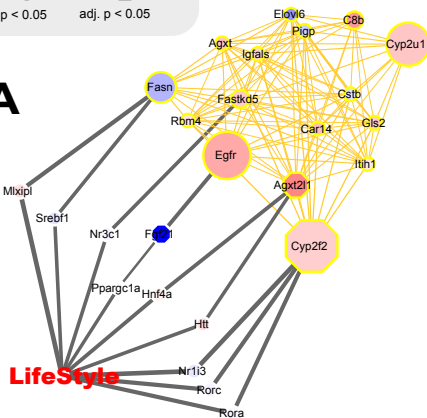**B**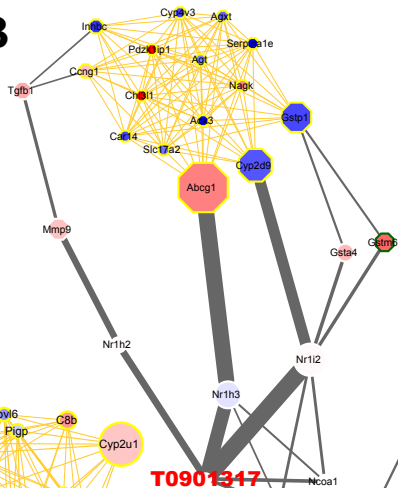**C**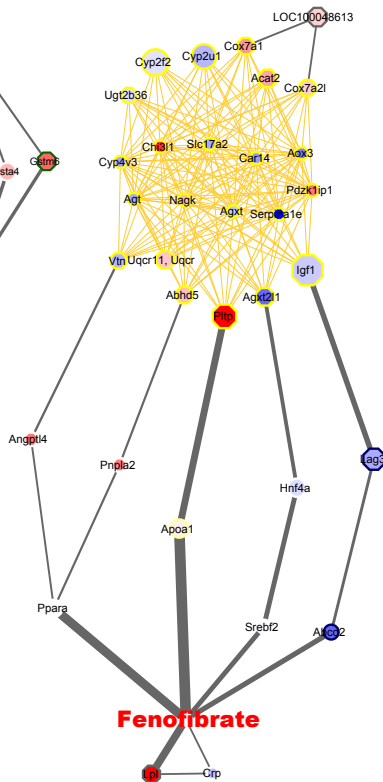

Supplement: Additional file 7: Figure S3. — Network visualization of a subnetwork underlying the signature for module A, see Figure 4 for detailed legend. [file s12918-014-0108-0-S7.pdf]
