# Supplementary material for: Network signatures link hepatic effects of anti-diabetic interventions with systemic disease parameters
Source: BMC Syst Biol. 2014 Sep 11;8:108. doi: 10.1186/s12918-014-0108-0 (PMC4363943; doi:10.1186/s12918-014-0108-0)
Supplement: Additional file 3: — Module selection analysis. This document contains a detailed description of the analysis on all co-expression modules in order to select those relevant for further analysis. [file s12918-014-0108-0-S3.pdf]

Annotation of co-expression modules to biological function

To identify common biological functions of the genes within each module, overrepresentation analysis on the Gene Ontology (GO) was performed. For 10 out of 14 modules, a significant overrepresentation (adjusted p-value < 0.01) in at least one GO term was found (Table 1). The module for which the most significantly enriched GO terms were identified was module B, annotated to immune and inflammation related functions. Metabolic process related GO terms were enriched for module genes from module A, module C (lipid and acyl-CoA metabolism), and module M (electron transport chain). The term "regulation of primary metabolic processes" was enriched with genes from module H, implying a regulatory role for the proteins encoded by the genes in this module. The module G was enriched for circadian rhythm genes, in concordance with previous findings that hepatic circadian rhythm gene expression is strongly affected by HFD [1].

Correlation of co-expression to disease parameters

For the 14 modules with a valid eigengene, correlations were calculated with each of the 16 disease parameters measured in the ADT dataset. In total, 10 significant correlations between modules and disease parameters were found ($|r| > 0.75$, $p < 1E-7$, Table 1). These correlations include 4 co-expression modules (A, B, C, E) and 4 different parameters (liver weight, atherosclerosis, cholesterol, and triglycerides), with much overlap between these disease parameters (Supplementary figure 3-1, see below).

The disease parameters related to dyslipidemia, which were previously found to be deteriorated after both drug interventions (plasma and intrahepatic triglycerides, cholesterol, atherosclerotic lesion area, liver weight) correlated with multiple co-expression modules. In contrast, although glycemia / insulin sensitivity related disease parameters (glucose, insulin, QUICKI) and obesity (body weight, epididymal fat weight) are fully resolved by T0901317 and partly by fenofibrate [1], no co-expression module correlated with these parameters. This indicates that hepatic target activation determines changes in dyslipidemia rather than disglycemia.

Selection of co-expression modules driven by interventions

Since the co-expression network was generated based on data from all intervention groups (HFD, DLI, T0901317 and fenofibrate) there is a possibility that some interventions determine the correlations within the module more than others. To investigate which modules are driven by which treatments, we performed over-representation analysis of differentially expressed genes (DEGs) relative to the control (HFD) group (Supplementary table 3-1, see below). An enrichment with DEGs for a given intervention indicates that the gene expression in the module is affected by that intervention and thus that the module may be relevant for explaining the link between the intervention target and disease parameter.

Several modules showed a distinct enrichment for specific interventions, such as two modules that were enriched for DLI but not drug interventions (E, G), modules enriched for both drug interventions but not DLI (D, M), and modules enriched specifically for T0901317 (J, L), or fenofibrate (K). Interestingly, several modules were enriched specifically for DEGs of drug interventions and not for DEGs of HFD

(D, J, L, M), which indicates the underlying mechanisms are not inherent to the disease induced by HFD but are nevertheless affected by drug intervention. For example, module M is enriched for genes for which the protein products participate in the electron transport chain, which are expected to increase especially after intervention with both fenofibrate and T0901317 [2], as result of increased hepatic fatty acid oxidation. Three modules (H, I, F) were not enriched for any of the diet or drug interventions. These modules also showed very poor correlations with any of the disease parameters, and their gene co-expression may be due to confounding factors not directly related to the disease process.

In addition to over-representation analysis, the DEGs for interventions were split into three groups indicating genes for which the expression is either reversed compared to HFD condition (changed in HFD vs chow and changed in opposite direction for intervention vs HFD), regulated in the same direction, i.e. deregulated compared to HFD condition (changed in HFD vs chow, and in same direction for intervention vs HFD), or specific for the intervention (not changed in HFD vs chow, but changed in intervention vs HFD). This provides insight in which intervention may revert, aggravate, or introduce additional deregulation of module genes.

The results for the three modules with significant GO annotation and correlation to a disease parameter (A, B, C) are shown in Table 2. All three modules are enriched for genes differentially expressed after HFD, which are largely reversed by DLI. This is in concordance with the observed improvement by DLI of the disease parameters correlating with these modules. In contrast, the drug interventions further deregulate nearly all genes in the module that were also regulated by HFD. These opposite effects match the observed deterioration of the corresponding disease parameters by both drug interventions. In addition, the modules show a large part of additional genes regulated by drugs that were not deregulated by disease, indicating that the drug interventions target or result in different metabolic and immune-related mechanisms than those related to disease progression.

Module B, annotated to immune response and inflammation related processes, was most strongly enriched with DEGs for the T0901317 intervention. Notably, the majority of DEGs (141 out of 146) in the module were upregulated by T0901317 compared to HFD and show the opposite response for DLI where these are downregulated compared to HFD. These genes include genes encoding for several macrophage markers (CD14, CD68, LYZ), and immune cell specific proteins (CD86, CD74, CD83, CD52, CD53, Rac2).

Tables & Figures

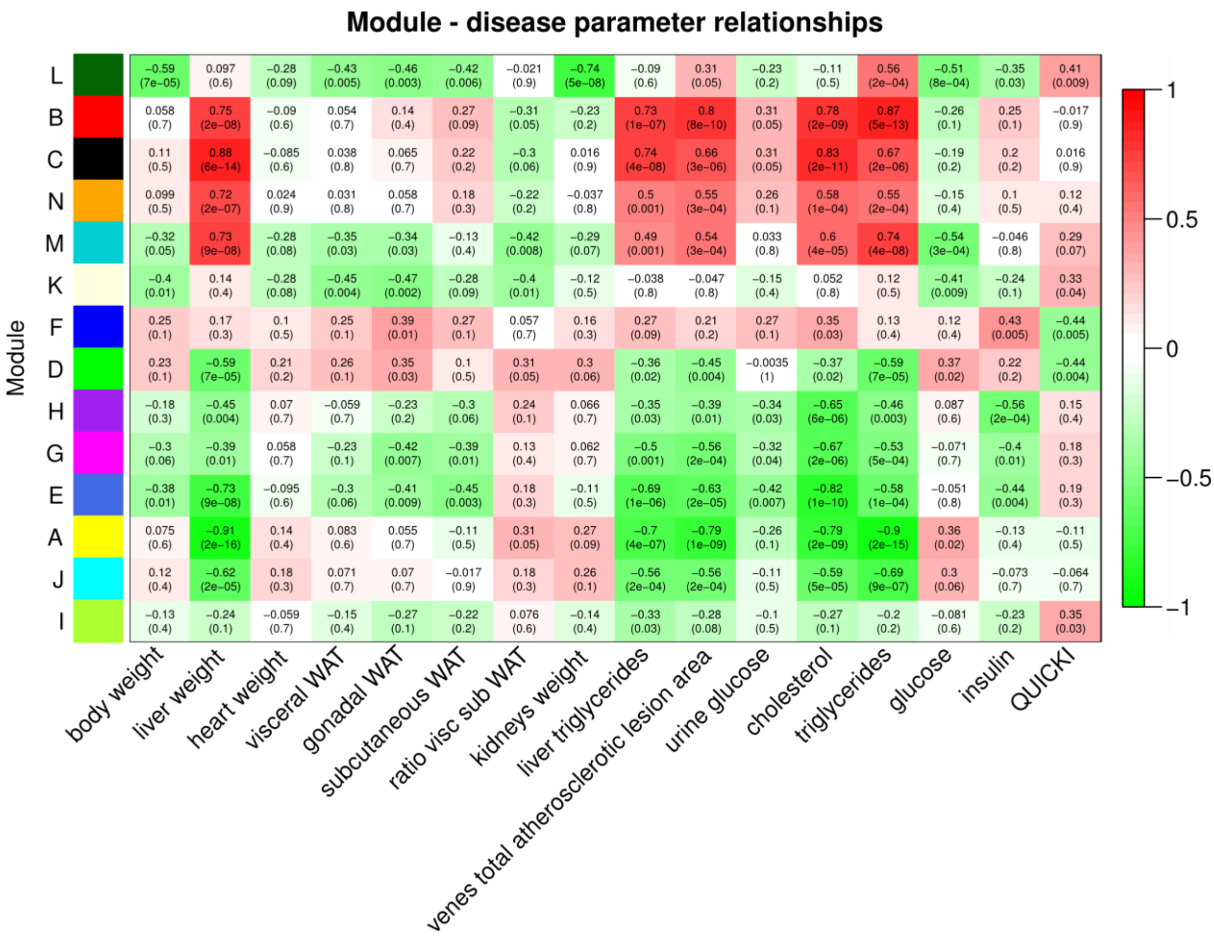

Supplementary Figure 3-1: Heatmap of the correlations between all modules with a valid eigengene (rows) and all disease parameters (columns). Each cell displays the correlation coefficient and corresponding p-value. Each cell is shaded by correlation coefficient, where red is positive correlation and green is negative correlation.

treatment	moduleSize	moduleDeg	pvalue	reversed	deregulated	additional	reversed %	deregulated %	additional %
**D (green)**									
16wkHighFat	185	16	0,998804						
LifeStyle	185	19	0,743861	12	0	7	63,15789	0	36,84211
Fenofibrate	185	62	2,95E-06	9	2	51	14,51613	3,225806	82,25806
T0901317	185	121	2,82E-29	13	3	105	10,7438	2,479339	86,77686
**B (red)**									
16wkHighFat	161	79	5,32E-23						
LifeStyle	161	77	9,62E-31	59	0	18	76,62338	0	23,37662
Fenofibrate	161	31	0,539475	1	9	21	3,225806	29,03226	67,74194
T0901317	161	146	7,58E-68	1	72	73	0,684932	49,31507	50
**J (cyan)**									
16wkHighFat	83	4	0,999591						
LifeStyle	83	9	0,633054	0	0	9	0	0	100
Fenofibrate	83	26	0,005977	1	0	25	3,846154	0	96,15385
T0901317	83	71	9,32E-30	4	0	67	5,633803	0	94,3662
**N (orange)**									

16wkHighFat	22	13	4,91E-06						
LifeStyle	22	0	1	0	0	0	NaN	NaN	NaN
Fenofibrate	22	8	0,046651	0	3	5	0	37,5	62,5
T0901317	22	15	4,59E-05	0	8	7	0	53,33333	46,66667
**A (yellow)**									
16wkHighFat	197	49	0,000726						
LifeStyle	197	46	1,98E-06	26	0	20	56,52174	0	43,47826
Fenofibrate	197	157	2,36E-76	0	36	121	0	22,92994	77,07006
T0901317	197	194	1,03E-109	0	48	146	0	24,74227	75,25773
**E (royalblue)**									
16wkHighFat	51	43	5,49E-27						
LifeStyle	51	26	4,82E-12	26	0	0	100	0	0
Fenofibrate	51	17	0,012487	0	13	4	0	76,47059	23,52941
T0901317	51	11	0,821625	1	7	3	9,090909	63,63636	27,27273
**H (purple)**									
16wkHighFat	118	9	0,997686						
LifeStyle	118	10	0,888987	7	0	3	70	0	30
Fenofibrate	118	35	0,004349	2	1	32	5,714286	2,857143	91,42857
T0901317	118	27	0,827919	2	0	25	7,407407	0	92,59259
**I (greenyellow)**									
16wkHighFat	112	3	0,999999						
LifeStyle	112	10	0,848432	1	0	9	10	0	90
Fenofibrate	112	4	1	0	0	4	0	0	100
T0901317	112	2	1	0	0	2	0	0	100
**K (lightyellow)**									
16wkHighFat	54	29	1,67E-10						
LifeStyle	54	5	0,763991	5	0	0	100	0	0
Fenofibrate	54	28	8,29E-08	21	0	7	75	0	25
T0901317	54	16	0,335	12	0	4	75	0	25
**G (magenta)**									
16wkHighFat	125	92	4,26E-47						
LifeStyle	125	56	6,13E-21	54	0	2	96,42857	0	3,571429
Fenofibrate	125	7	0,999998	5	2	0	71,42857	28,57143	0
T0901317	125	10	1	2	4	4	20	40	40
**C (black)**									
16wkHighFat	142	59	2,04E-13						
LifeStyle	142	52	4,99E-15	40	0	12	76,92308	0	23,07692
Fenofibrate	142	125	3,55E-71	0	51	74	0	40,8	59,2
T0901317	142	123	4,24E-52	1	50	72	0,813008	40,65041	58,53659
**F (blue)**									
16wkHighFat	369	21	1						
LifeStyle	369	40	0,695422	13	0	27	32,5	0	67,5
Fenofibrate	369	9	1	0	0	9	0	0	100
T0901317	369	21	1	5	1	15	23,80952	4,761905	71,42857
**L (darkgreen)**									
16wkHighFat	41	13	0,008985						

LifeStyle	41	12	0,001744	9	0	3	75	0	25
Fenofibrate	41	15	0,007263	0	4	11	0	26,66667	73,33333
T0901317	41	35	3,25E-15	13	0	22	37,14286	0	62,85714
**M (darkturquoise)**									
16wkHighFat	39	13	0,005637						
LifeStyle	39	7	0,157731	5	0	2	71,42857	0	28,57143
Fenofibrate	39	36	7,58E-23	13	0	23	36,11111	0	63,88889
T0901317	39	39	1,91E-23	13	0	26	33,33333	0	66,66667

Supplementary table 3-1: Results of the over-representation analysis of differentially expressed genes (DEGs) relative to the control (HFD) group in the modules. The same as table 2, but showing results for all modules with a valid eigengene.

References:

1. Radonjic M, Wielinga PY, Wopereis S, Kelder T, Goelela VS, Verschuren L, Toet K, van Duyvenvoorde W, van der Werff van der Vat B, Stroeve JHM, Cnubben N, Kooistra T, van Ommen B, Kleemann R: **Differential Effects of Drug Interventions and Dietary Lifestyle in Developing Type 2 Diabetes and Complications: A Systems Biology Analysis in LDLr−/− Mice**. *PLoS One* 2013, **8**:e56122.

2. Hu T, Foxworthy P, Siesky A, Ficorilli J V, Gao H, Li S, Christe M, Ryan T, Cao G, Eacho P, Michael MD, Michael LF: **Hepatic peroxisomal fatty acid beta-oxidation is regulated by liver X receptor alpha.** *Endocrinology* 2005, **146**:5380–7.
